# Uncoordinated Loss of Chromatid Cohesion Is a Common Outcome of Extended Metaphase Arrest

**DOI:** 10.1371/journal.pone.0022969

**Published:** 2011-08-02

**Authors:** Deanna Stevens, Reto Gassmann, Karen Oegema, Arshad Desai

**Affiliations:** Ludwig Institute for Cancer Research and Department of Cellular and Molecular Medicine, University of California San Diego, La Jolla, California, United States of America; Virginia Tech, United States of America

## Abstract

Chromosome segregation requires coordinated separation of sister chromatids following biorientation of all chromosomes on the mitotic spindle. Chromatid separation at the metaphase-to-anaphase transition is accomplished by cleavage of the cohesin complex that holds chromatids together. Here we show using live-cell imaging that extending the metaphase bioriented state using five independent perturbations (expression of non-degradable Cyclin B, expression of a Spindly point mutant that prevents spindle checkpoint silencing, depletion of the anaphase inducer Cdc20, treatment with a proteasome inhibitor, or treatment with an inhibitor of the mitotic kinesin CENP-E) leads to eventual scattering of chromosomes on the spindle. This scattering phenotype is characterized by uncoordinated loss of cohesion between some, but not all sister chromatids and subsequent spindle defects that include centriole separation. Cells with scattered chromosomes persist long-term in a mitotic state and eventually die or exit. Partial cohesion loss-associated scattering is observed in both transformed cells and in karyotypically normal human cells, albeit at lower penetrance. Suppressing microtubule dynamics reduces scattering, suggesting that cohesion at centromeres is unable to resist dynamic microtubule-dependent pulling forces on the kinetochores. Consistent with this view, strengthening cohesion by inhibiting the two pathways responsible for its removal significantly inhibits scattering. These results establish that chromosome scattering due to uncoordinated partial loss of chromatid cohesion is a common outcome following extended arrest with bioriented chromosomes in human cells. These findings have important implications for analysis of mitotic phenotypes in human cells and for development of anti-mitotic chemotherapeutic approaches in the treatment of cancer.

## Introduction

Accurate passage through mitosis is a highly orchestrated process that relies on the timely coordination of multiple events to ensure proper segregation of genetic material. Errors in chromosome segregation lead to aneuploidy, a well-known hallmark of human cancers. A key mechanism that ensures each daughter cell receives the correct chromosome content is the maintenance of the physical links between sister chromatids until all chromosomes become properly bioriented on the mitotic spindle [1]. Sister chromatids are held together by cohesin, a multisubunit protein complex that is loaded along the length of each pair concomitant with replication in S phase [2]. A majority of the cohesin resides on the chromosome arms and is removed at the beginning of mitosis, whereas centromeric cohesin remains bound until the metaphase-to-anaphase transition [3]. The prophase removal of cohesin involves the activity of the kinases Plk1 and Aurora B [4], [5] as well as the physical interaction of the protein Wapl with the cohesin complex [6], [7]. In contrast, the removal of cohesin at the onset of anaphase requires the protease separase, which cleaves the cohesin subunit Scc1 [8], [9]. Separase is activated at anaphase onset when the anaphase promoting complex/cyclosome (APC/C), an E3 ubiquitin ligase, targets its inhibitor securin for degradation and reduces Cdk1 activity [10], [11]. The APC/C activity targeting securin is inhibited by the spindle assembly checkpoint until all chromosomes are fully aligned on the metaphase plate. When the last pair of chromatids properly aligns, the checkpoint is turned off, which leads to APC/C-mediated degradation of securin, and in turn activates separase. Separase then cleaves the centromeric cohesin in a coordinated manner so that cohesin is lost from all sister chromatids as the cell enters anaphase. Previous studies have investigated the consequences of uncoupling these regulated events and have shown how important their coordination is for proper chromosome segregation and progression through mitosis [12], [13], [14].

Under conditions where the checkpoint signal is sustained in the presence of fully aligned chromosomes, cells persist in mitosis for a variable amount of time before the metaphase plate begins to break down. This phenotype, termed chromosome scattering, was initially described as a result of inhibition of the spindle and kinetochore associated protein Ska3 [15] and was later observed in cells expressing a point mutant of the kinetochore protein Spindly [16]. Since both perturbations cause the checkpoint to remain active without interfering with chromosome alignment, we hypothesized that chromosome scattering is not perturbation specific, but rather a general result of prolonged arrest in metaphase. In the present study we set out to determine how frequently cells scatter their chromosomes after a persistent arrest in a relatively unperturbed mitosis and to investigate the factors contributing to this phenotype. Using both live cell and fixed image analyses we found chromosome scattering to occur in a wide variety of conditions that prevent exit from mitosis with a completely or near-completely aligned metaphase plate. Cells with scattered chromosomes had partial loss of chromatid cohesion and defects in spindle structure. Scattering was inhibited by either stabilizing microtubules or by preventing the removal of cohesion. These results establish that uncoordinated loss of chromatid cohesion is a common outcome of extended metaphase arrest in human cells.

## Results

### Prolonged Metaphase Arrest Induced by Five Distinct Treatments Leads to Chromosome Scattering

**Figure 1:**
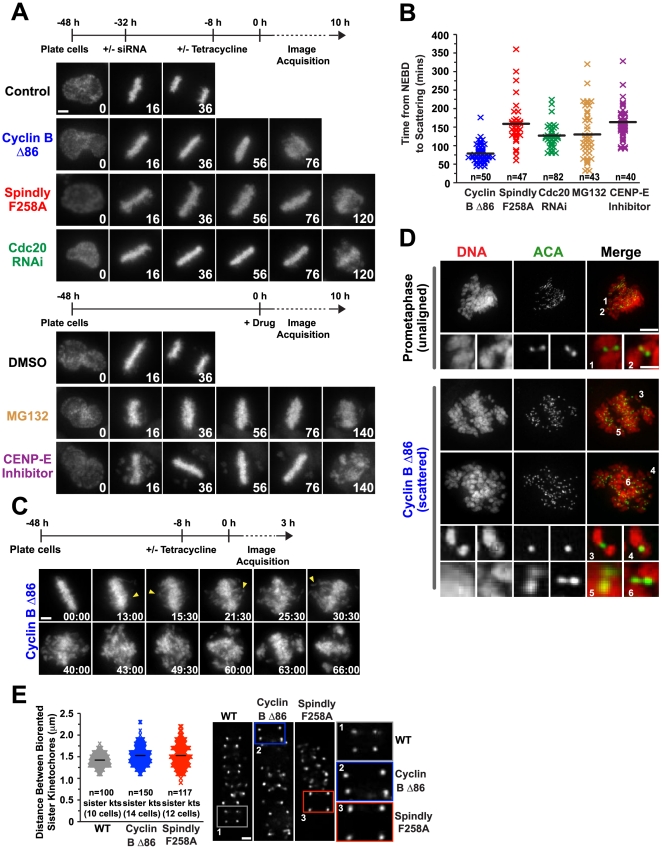
Chromosome scattering results from a prolonged metaphase arrest and is characterized by uncoordinated loss of chromatid cohesion. (**A**) Selected images from time-lapse imaging sequences of cells expressing histone H2b:mRFP. Time (in mins) is indicated on the lower right of each panel; time 0 indicates NEBD. The scheme for each set of experiments is depicted above the images. Both Cyclin B Δ86 and the Spindly motif mutant are under regulated expression, induced by Tetracycline. For Spindly F258A, endogenous Spindly was knocked down via RNAi prior to expression of the mutant. Scale bar, 5 µm. (**B**) Quantification of the experiments in (A) for the time cells spent in mitosis prior to scattering. Each mark represents one cell and the black line indicates the average. For MG132, the average time spent in mitosis was calculated from metaphase alignment to scattering since no new cells entered mitosis after addition of the drug and a majority of the cells were fully aligned at the beginning of filming. (**C**) Selected images from a time-lapse imaging sequence of cells expressing histone H2b:mRFP. Images were acquired every 30 seconds, time (in mins:secs) is indicated on the lower right of each panel; time 0 represents the fully aligned metaphase plate just prior to the onset of visible scattering. Arrowheads indicate chromatids that have escaped from the metaphase plate. Scale bar, 5 µm. (**D**) Immunofluorescence images of cells that are either unaligned in prometaphase or have scattered their chromosomes, stained for DNA and centromere marker ACA. Paired sister chromatids are seen off the plate in prometaphase, whereas single chromatids are seen off the plate in scattered cells. Scale bar, 5 µm; magnified image 1 µm. (**E**) Distance between the ACA signals at sister kinetochores at metaphase for the indicated conditions. Each mark represents one pair of sister kinetochores, and the black line indicates the average. Immunofluorescence of a single Z plane of a metaphase cell stained for ACA for each condition is shown highlighting the difference in interkinetochore distance. Scale bar, 1 µm.

To assess whether chromosome scattering is a general consequence of a prolonged metaphase arrest, we tested whether this phenotype is induced by multiple perturbations used to hold cells in a metaphase-like state. For this purpose, HeLa FRT cells (harboring a single FRT recombination site that was either empty or integrated with tetracycline-regulated transgenes expressing either the Spindly F258A or Cyclin B Δ86 mutants [16]) expressing fluorescent histone H2b were exposed to five distinct conditions that prevent anaphase entry. Chromosomes aligned fully with normal kinetics in four of these conditions—expression of non-degradable Cyclin B Δ86 [17], [18], expression of a Spindly F258A mutant following endogenous Spindly depletion, Cdc20 depletion, or proteasome inhibitor treatment; in the fifth condition, treatment with a CENP-E kinesin inhibitor [19], [20] the majority of chromosomes aligned but a few persisted at the poles. Live imaging revealed that chromosome scattering is eventually observed for the aligned chromosomes with all five perturbations (Fig. 1A; Movie S1 and S2). In four out of the five conditions, 100% of the cells exhibited scattering. Following Cdc20 knockdown, half of the cells that entered mitosis scattered their chromosomes; the remainder progressed through mitosis with a mild delay (51±30 mins from NEBD to anaphase compared to 34±5 mins for the control), most likely due to partial penetrance of the RNAi-mediated knockdown [21]. The time from NEBD to scattering was variable between the different conditions, ranging from 44 to 360 mins (Fig. 1B). Chromosomes remained condensed during the scattered state indicating a persistent mitotic arrest; for the Spindly mutant, prior work showed persistence of securin and Cyclin B in the scattered state [16]. We also observed securin and Cyclin B persistence in scattered cells knocked down for Cdc20 or treated with the CENP-E inhibitor (Fig. S1A and B). Scattered cells expressing Cyclin B Δ86 exhibited high securin levels (Fig. S1B), indicating that the single copy Cyclin B Δ86 insertion does not robustly activate the anaphase promoting complex and trigger an anaphase-like state with high Cdk1 kinase activity [17], [18]. We did observe a small proportion (∼10%) of the cells expressing Cyclin B Δ86 that were in an anaphase-like state with reduced securin levels (Fig. S1C); these cells are morphologically distinct from the scattered cells, as they show equal separated chromosome masses, and were not included in analysis of the scattered state.

These observations indicate that when progression into anaphase is prevented in HeLa FRT cells, chromosome scattering is a frequent outcome irrespective of the precise means used to prevent anaphase entry.

### Chromosome Scattering is Initiated by Uncoordinated Loss of Sister Chromatid Cohesion

We next investigated the scattering phenotype at higher resolution using both live imaging and fixed analysis. High-resolution live imaging revealed initial dispersion of a few fluorescent H2b masses from the metaphase plate, followed by increasing dispersion of chromosomes and rotation of the spindle within the cell (Fig. 1C; Movie S3). High-resolution fixed analysis revealed the frequent presence of single chromatids in the scattered state, indicating loss of sister chromatid cohesion on some of the chromosomes in the cell. In the same cells, sisters at the spindle equator remained paired (Fig. 1D). This result suggests that scattering induced loss of cohesin is uncoordinated, in contrast to anaphase onset in which cohesin is removed from all sister chromatids simultaneously.

The above observations suggest that partial and uncoordinated loss of chromatid cohesion is the trigger for entry into the scattered state. To assess if weakened cohesion could be detected prior to the onset of scattering, we analyzed the inter-kinetochore distance in cells with fully aligned chromosomes prior to visible scattering for arrests generated using Cyclin B Δ86 or the Spindly F258A mutant. Control cells were mock transfected and treated with tetracycline in parallel. In this experiment, which involved fixed analysis of an asynchronous population of cells, each cell analyzed had spent a variable and undetermined amount of time with fully aligned chromosomes. A mild but statistically significant increase is observed for the average inter-kinetochore distance for the two perturbations (unpaired *t* test, p = 0.0001 for Cyclin B Δ86 and 0.0005 for the Spindly F258A mutant when compared to control). However, we observed the presence of a number of hyper-stretched sister kinetochores in cells expressing either the Spindly F258A or Cyclin B Δ86 mutants (Fig. 1E), which are not detected in the control cells. We suggest this extended stretch reflects weakened sister cohesion that is less able to resist the outward pulling forces generated on the kinetochores and will soon after lead to dispersion of chromatids from the metaphase plate and entry into the scattered state. These measurements support the notion that uncoordinated loss of cohesion underlies the scattering phenotype.

### Spindle Defects are a Secondary Consequence of Entry into the Scattered State

**Figure 2:**
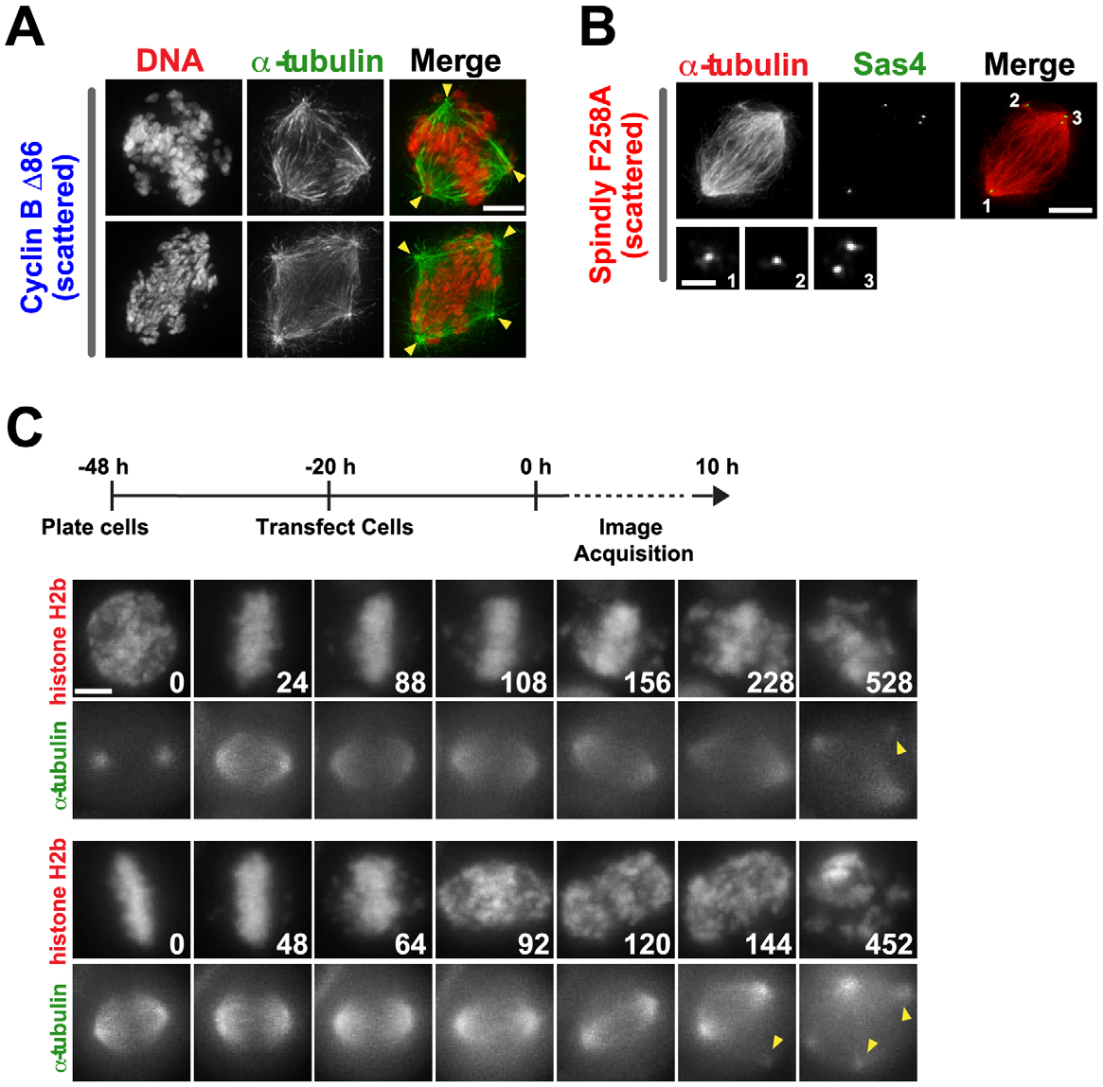
Chromosome scattering results in spindle defects that are secondary to loss of cohesion. (**A, B**) Immunofluorescence images of scattered cells stained with DNA and (A) α-tubulin [arrowheads denote all spindle poles] and (B) centriole marker Sas4. Scale bar, 5 µm; magnified image 1 µm. (**C**) Selected images from time-lapse imaging sequences of cells expressing mRFP:histone H2b and YFP:α-tubulin. The experimental scheme is depicted above the images. Time (in mins) is indicated on the lower right of each panel; time 0 for panel set 1 is NEBD and for panel set 2 full alignment in metaphase. Arrowheads indicated the appearance of extra spindle poles. Scale bar, 5 µm.

Analysis in fixed cells revealed that the scattered state is frequently correlated with spindle defects that include extra spindle poles and disengaged centrioles (Fig. 2A and B). Live imaging of a HeLa cell line expressing mRFP:histone H2b and YFP:α-tubulin that was transfected to transiently express nondegradable Cyclin B Δ86 revealed that chromosomes scattered from the plate prior to the emergence of spindle defects and spindle rotation (Fig. 2C, n = 14 cells). The time in which spindle defects arose after cells scattered their chromosomes was variable, ranging from 60 to 344 mins after the first visible sign of scattering. In 5/6 cells where spindle rotation was clearly visible, rotation preceded the appearance of extra spindle poles. In fixed analysis, the spindle rotation phenotype appears as spindle malorientation relative to the cell substrate. These results suggest that partial and uncoordinated loss of chromatid cohesion is the initiating event in scattering, followed by subsequent spindle defects.

### Karyotypically Normal Human Cells are Susceptible to Chromosome Scattering After An Extended Metaphase Arrest

**Figure 3:**
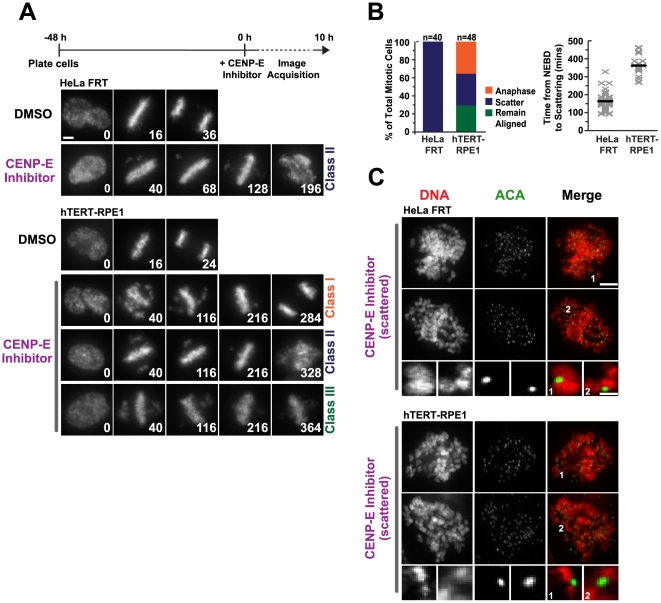
Chromosome scattering occurs in karotypically normal cells. (**A**) Selected images from time-lapse imaging sequences of cells expressing histone H2b:mRFP for HeLa FRT and hTERT-RPE1 cells treated with a CENP-E inhibitor. Time (in mins) is indicated on the lower right of each panel; time 0 is NEBD. The experimental scheme is depicted above the images. Scale bar, 5 µm. (**B**) Comparison and quantification of the cellular response to the CENP-E inhibitor. (**C**) Immunofluorescence images of scattered cells that are stained for DNA and centromere marker ACA. Individual chromatids are seen off the plate in the scattered state for both cells lines treated with the CENP-E inhibitor. Scale bar, 5 µm; magnified image 1 µm.

The observation that entry into the chromosome scattering state is a frequent response to a prolonged metaphase arrest in HeLa FRT cells prompted us to test whether this phenotype is also observed in a karyotypically normal human cell line. For this purpose, we analyzed telomerase-immortalized RPE1 cells (hTERT-RPE1) expressing fluorescent histone H2b. We initially attempted Cdc20 RNAi in these cells to generate a metaphase arrest but low transfection efficiency prevented significant knockdown and extended arrest in metaphase. We therefore treated hTERT-RPE1 cells with the CENP-E inhibitor and imaged them over time (Fig. 3A). Of the treated cells, 35% exhibited chromosome scattering following an extended arrest (Class II), 35% delayed in mitosis and progressed into anaphase (Class I) and 30% remained arrested in mitosis for the duration of the 10 hr time lapse without scattering, but did exhibit a large number of polar chromosomes that had never congressed to the metaphase plate (Class III). When compared to HeLa FRT cells, the Class II hTERT-RPE1 cells took over twice as long to scatter their chromosomes (362±62 mins after NEBD for hTERT-RPE1 and 164±50 mins for HeLa FRT, Fig. 3B). Fixed analysis of both cell lines treated with the CENP-E inhibitor revealed the presence of single chromatids in cells with scattered chromosomes (Fig. 3C), confirming the idea that scattering results from the uncoordinated loss of cohesion.

These results establish that scattering is also observed at a significant frequency in karyotypically normal human cells following an extended metaphase arrest, albeit at lower penetrance and after a longer arrest time than in the highly aneuploid HeLa FRT cells.

### The Spindle Checkpoint is Reactivated in the Scattered State and Leads to Long-Term Persistence in Mitosis

**Figure 4:**
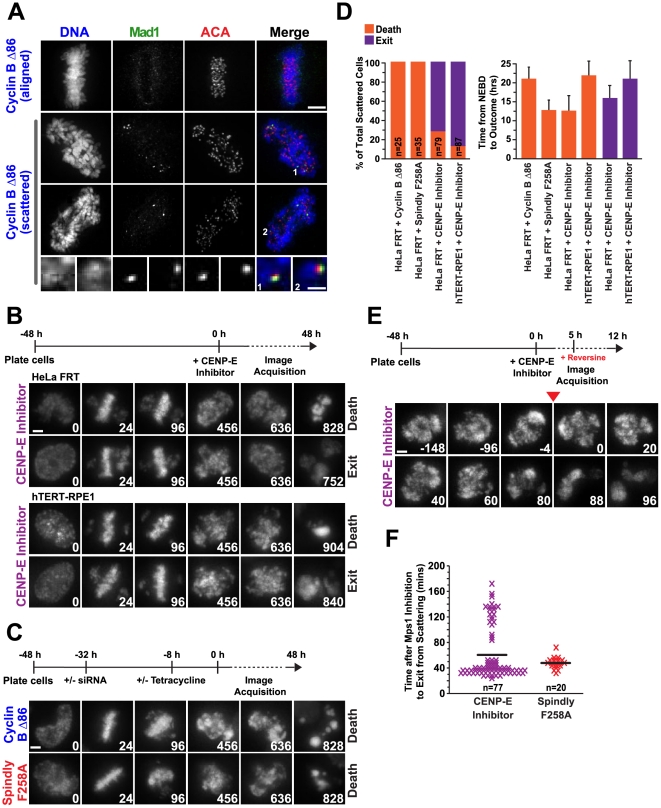
The spindle assembly checkpoint is reactivated upon loss of cohesion and is required to maintain the scattered state. (**A**) Immunofluorescence images of metaphase-aligned and scattered cells expressing Cyclin B Δ86 stained for DNA, Mad1 and ACA. No Mad1 is seen on fully aligned chromosomes, whereas Mad1 relocalizes to kinetochores of single chromatids upon scattering. Scale bar, 5 µm; magnified image 1 µm. (**B and C**) Selected images from time-lapse imaging sequences of cells expressing histone H2b:mRFP for (B) HeLa FRT and hTERT-RPE1 cells treated with a CENP-E inhibitor and (C) HeLa FRT cells expressing either Cyclin B Δ86 or the Spindly motif mutant. The scheme for each set of experiments is depicted above the images. Time (in mins) is indicated on the lower right of each panel; time 0 is NEBD. Scale bar, 5 µm. (**D**) Determination and quantification of the endpoint of cells in the scattered state. All error bars represent SD. (**E**) Selected images from time-lapse imaging sequences of cells expressing histone H2b:mRFP. HeLa FRT cells were imaged for 5 hours in the presence of the CENP-E inhibitor prior to the addition of 0.5 µM reversine. The red arrowhead indicates the frame after which reversine was added for that cell. Time (in mins) is indicated in the lower right of each panel; time 0 is directly after drug addition. Scale bar, 5 µm. (**F**) Quantification of the results for (E). Each mark represents one cell and the black line indicates the average.

The spindle checkpoint is silenced by removal of checkpoint proteins from the kinetochore following microtubule attachment [22]. Uncoordinated loss of chromatid cohesion is expected to reactivate the checkpoint on the individual chromatids that are no longer stably attached to the spindle. Consistent with this, we observed Mad1 labeling on kinetochores of single chromatids in scattered Cyclin B Δ86-expressing cells (no Mad1 labeling is observed on metaphase aligned chromosomes prior to scattering onset, Fig. 4A).

We next assessed the long-term consequences of entry into the scattered state. For this purpose, we filmed HeLa FRT cells or hTERT-RPE1 cells for 48 hrs following treatment with the CENP-E inhibitor. For both cell lines, the scattered cells persisted in their aberrant state for 10–25 hrs and then exhibited one of two fates: mitotic exit (characterized by chromosome decondensation and separation of the chromatin into two or three distinct masses) or death (characterized by the presence of hypercondensed chromatin masses) (Fig. 4B). For both cell lines, the predominant long-term outcome of chromosome scattering via inhibition of CENP-E was mitotic exit. A portion of the cells that exited the scattering state subsequently died in interphase (50% for HeLa FRT cells and 4% for hTERT-RPE1 cells); the remaining population of cells neither died nor entered another round of mitosis during the 48 hr filming period.

We also filmed HeLa FRT cells expressing the Spindly F258A mutant or Cyclin B Δ86 for 48 hrs. For these two conditions, which lock the spindle checkpoint into an active state or prevent mitotic exit, respectively [16], [18] cells persisted with condensed, scattered chromosomes and eventually died 10–25 hrs after initial entry into mitosis (Fig. 4C).

We next assessed whether the long-term persistence in the scattered state required spindle checkpoint activity. We first treated HeLa FRT cells with the CENP-E inhibitor and filmed them for 5 hrs. We then added the Mps1 inhibitor reversine [23] and assessed the outcome in cells that had already been in a scattered state for 1–5 hrs. We also performed a similar experiment with scattered cells generated using the Spindly F258A mutant. Without reversine addition, scattered cells generated by either perturbation spent 8–14 hrs in mitosis before exiting or undergoing cell death (Fig. 4D). For both perturbations, treatment with reversine led to rapid exit from the scattered mitotic state, as determined by chromosome decondensation (Fig. 4E and F). This result, together with the presence of checkpoint proteins on the kinetochores of scattered chromatids, suggests that spindle checkpoint activity maintains the cells in the long-term scattered mitotic state.

Overall, the long-term filming revealed that once cells enter the scattered state, they persist in this state for minimally 8 hrs and either exit in a non anaphase-like manner or die in the scattered state. In the cells that do exit the scattered state interphase cell death is frequently observed. The long-term persistence of this aberrant mitotic state is likely due to activation of the spindle checkpoint following partial loss of chromatid cohesion. Given that, under our conditions, normal mitotic duration (from NEBD to anaphase) in HeLa FRT cells is ∼34 mins and in hTERT-RPE1 cells is ∼29 mins, the persistence of the scattered state for over 10–25 hrs indicates that, once cells scatter their chromosomes, they are effectively in a terminal state and are unable to recover and proliferate.

### Prolonged Biorientation With Dynamic Microtubules Promotes Chromosome Scattering

**Figure 5:**
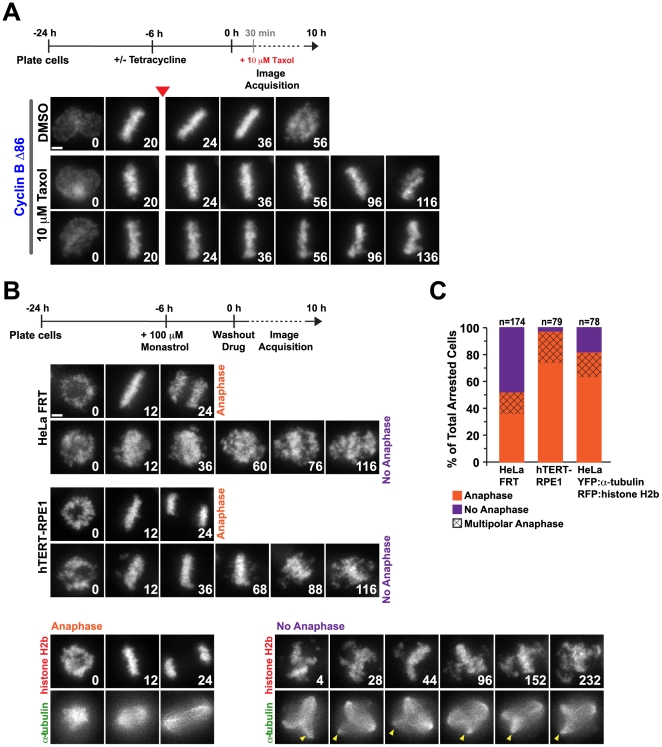
Biorientation with dynamic microtubule-dependent tension at kinetochores contributes to the onset of chromosome scattering. (**A**) Selected images from time-lapse imaging sequences of Cyclin B Δ86 HeLa FRT cells expressing histone H2b:mRFP. The experimental scheme is depicted above the images. Taxol was added to an asynchronous population of cells after the initial 30 minutes of filming. The red arrowhead denotes the frame after which the drug was added. Time (in mins) is indicated on the lower right of each panel; time 0 is NEBD. Scale bar, 5 µm. (**B**) Selected images from time-lapse imaging sequences of cells expressing H2b:mRFP (HeLa FRT and hTERT-RPE1) or mRFP:histone H2b and YFP:α-tubulin. The scheme used for monastrol addition and washout is indicated on top. Time (in mins) is indicated on the lower right of each panel; time 0 is the start of filming, directly after monastrol washout. The arrowhead indicates the persistence of an extra spindle pole. Scale bar, 5 µm. (**C**) Quantification of the experiments in (B).

Prior to the onset of chromosome scattering, cells spend a significant amount of time arrested with most, or all, chromosomes aligned on the metaphase plate. In bioriented chromosomes, pulling forces from the dynamic spindle microtubules bound to kinetochores are resisted by cohesion between the sisters, placing sister chromatids under tension [24]. To determine if the action of dynamic microtubules contributes to the chromosome scattering phenotype, taxol was added to cells expressing Cyclin B Δ86 following full alignment in metaphase in order to stabilize microtubules and decrease tension across sisters. Asynchronously cycling cells induced for Cyclin B Δ86 expression were imaged live and monitored for entry into mitosis and alignment of chromosomes. Taxol was added after 30 minutes; enough time to allow for completion of, but not significant arrest in, metaphase. Continued long-term live imaging of these cells revealed that addition of taxol effectively inhibits chromosome scattering. After the addition of taxol, cells expressing Cyclin B Δ86 remain arrested with a metaphase plate without scattering (Fig. 5A). After a variable amount of time, the plate did begin to breakdown and collapse, however this is due to the effect of taxol on spindle structure, as the same phenotype is observed in control cells not expressing Cyclin B Δ86 (data not shown).

Since the average time a cell spends in mitosis prior to scattering is at least twice as long as the normal mitotic duration, it is possible that the amount of time a cell stays in mitosis, not just the forces from dynamic microtubules, contributes to the onset of chromosome scattering. To address this possibility, HeLa FRT cells, hTERT-RPE1 cells and HeLa cells expressing mRFP:histone H2b and YFP:α-tubulin were treated with monastrol, which arrests cells in mitosis with very little to no tension across sisters (average interkinetochore distance as measured by ACA staining: 0.42±0.07 µm in cells treated with monastrol compared to 1.41±0.15 µm in untreated cells fully aligned in metaphase). The cells were incubated in the presence of the drug for 6 hrs, given fresh, drug-free media and subsequently imaged live for 10 hrs. The majority of hTERT-RPE1 cells entered anaphase after washout (Fig. 5B and C), indicating that extended time in mitosis without tension across sisters does not induce scattering in this cell line. The HeLa FRT cells, however, responded in one of two ways: 50% entered anaphase after washout whereas the other 50% remain arrested with what appear to be scattered chromosomes (Fig. 5B and C). Since a proportion of the cells that did progress through mitosis did so with multipolar spindles (three or four distinct chromatin masses segregating at anaphase), it appeared that spindle defects were arising during the prolonged drug treatment in the HeLa FRT cells. We speculated that in the 50% of these cells that did not progress into anaphase, spindle defects as opposed to uncoordinated cohesion loss, was responsible for the progression defect. To test this, we directly imaged spindles and chromosomes under the same experimental regime in a different HeLa line stably expressing mRFP:histone H2b and YFP:α-tubulin. In this line, we observed that at the time of washout 40% of the cells had more than two α-tubulin foci, indicating aberrant spindle formation. Of the cells with multipolar spindles, 50% failed to progress into anaphase (Fig. 5B and C), supporting the view that spindle defects can arise during prolonged treatment in monastrol and perturb progression after washout.

Overall, these results suggest that spending an extended time in mitosis without tension across sisters does not promote uncoordinated cohesion loss to the same extent as when tension is present. However, depending on the cell line, different levels of spindle defects may be present at the time of drug washout, which greatly decreases the frequency of normal progression into anaphase.

Cumulatively, the analysis of acute taxol treatment and monastrol treatment followed by washout suggests that uncoordinated cohesion loss during extended metaphase arrest is promoted by the action of dynamic microtubules pulling on bioriented kinetochores.

### Strengthening Cohesion Inhibits Chromosome Scattering

**Figure 6:**
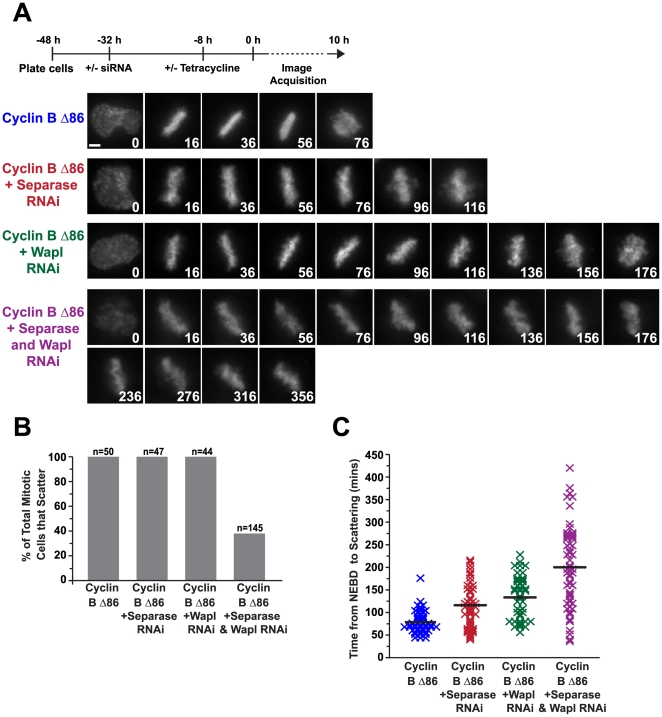
Chromosome scattering is prevented by inhibition of the two pathways that remove cohesion. (**A**) Selected images from time-lapse imaging sequences of Cyclin B Δ86 HeLa FRT cells expressing histone H2b:mRFP and subjected to the indicated perturbations. The experimental scheme is depicted on top. Time (in mins) is indicated on the lower right of each panel; time 0 is NEBD. Scale bar, 5 µm. (**B and C**) Quantification of the experiments in (A); (B) the percentage of total cells in mitosis that scatter for each condition and (C) the time that those cells remain in mitosis before scattering is plotted (only cells that scattered were analyzed for the double separase/Wapl inhibition); each mark represents one cell and the black line indicates the average.

The above results suggest that chromatid cohesion is lost in an uncoordinated manner during an extended mitotic arrest and this loss is promoted by dynamic microtubules exerting tension on the paired sisters. To test this model, we decided to strengthen cohesion by perturbing its cleavage during anaphase and/or its non-proteolytic removal during prophase. In an unperturbed mitosis, the majority of cohesin is removed in prophase by a non-proteolytic pathway involving the conserved Wapl protein and Polo-like kinase 1 [25], [26]. Centromeric cohesion is protected during prophase and removed at the metaphase-to-anaphase transition by the protease separase, whose activity is inhibited by the spindle assembly checkpoint until all of the chromosomes are properly bioriented [9]. We inhibited separase via RNAi in HeLa FRT cells expressing Cyclin B Δ86 (Fig. S2). Live imaging of these cells revealed that inhibition of separase led to a delay in the onset of chromosome scattering (NEBD to scattering: 119±51 mins with Cyclin B Δ86+separase RNAi versus 78±25 mins with Cyclin B Δ86 alone, Fig. 6A and C). However, all of the cells that entered mitosis scattered their chromosomes (Fig. 6B). In cells not expressing Cyclin B Δ86, separase knockdown caused two different phenotypes, 50% of the cells either progressed through anaphase, albeit with both a delay in timing (NEBD to anaphase onset: 51±12 mins versus 34±5 mins for the control) and the appearance of lagging chromosomes, whereas the other 50% remain arrested in mitosis with what appear to be scattered chromosomes (data not shown). Inhibition of Wapl by RNAi (Fig. S2) also led to a delay in the onset of chromosome scattering (NEBD to scattering: 134±47 mins with Cyclin B Δ86+Wapl RNAi, Fig. 6A and C). Similar to separase inhibition, all of the cells that entered mitosis with reduced Wapl function scattered their chromosomes (Fig. 6B). Reducing the function of both separase and Wapl together had a strongly synergistic effect. Only one-third of the cells that entered mitosis scattered their chromosomes in the double RNAi, whereas the remaining cells either died or persisted in mitosis with an observable metaphase plate (Fig. 6A and B). Of the cells that scattered, the time spent in mitosis prior to chromosome scattering increased nearly 3-fold (200±91 mins) compared to the control cells (78±25 mins) (Fig. 6C). 65% of the cells that persisted with a metaphase plate showed no signs of spindle rotation, as assessed by the orientation of the metaphase plate; the remaining 35% exhibited mild spindle rocking but not the full rotations observed in scattered cells. These results suggest that strengthening cohesion by blocking both cohesin removal pathways significantly inhibits the scattering phenotype.

## Discussion

In this study, we describe an aberrant mitotic state that is induced in karyotypically normal and abnormal human cells by multiple perturbations that prevent normal progression into anaphase. Entry into this state is triggered by partial and uncoordinated loss of sister chromatid cohesion on chromosomes aligned at the metaphase plate. Spindle checkpoint activation on the single chromatids leads to persistence in a mitotic state with increasing loss of cohesion and subsequent spindle defects, followed by an eventual non anaphase-like exit and/or cell death. These results suggest that chromatid cohesion is placed under stress in an attached bi-oriented state and extending this state can lead to cohesion failure. Discovery of this phenotype as a general result of prolonged metaphase arrest has implications for the interpretation of mitotic defects following perturbation of specific components and for the mechanistic analysis of anti-mitotic chemotherapeutic strategies.

### Distinction Between Scattering and Anaphase

**Figure 7:**
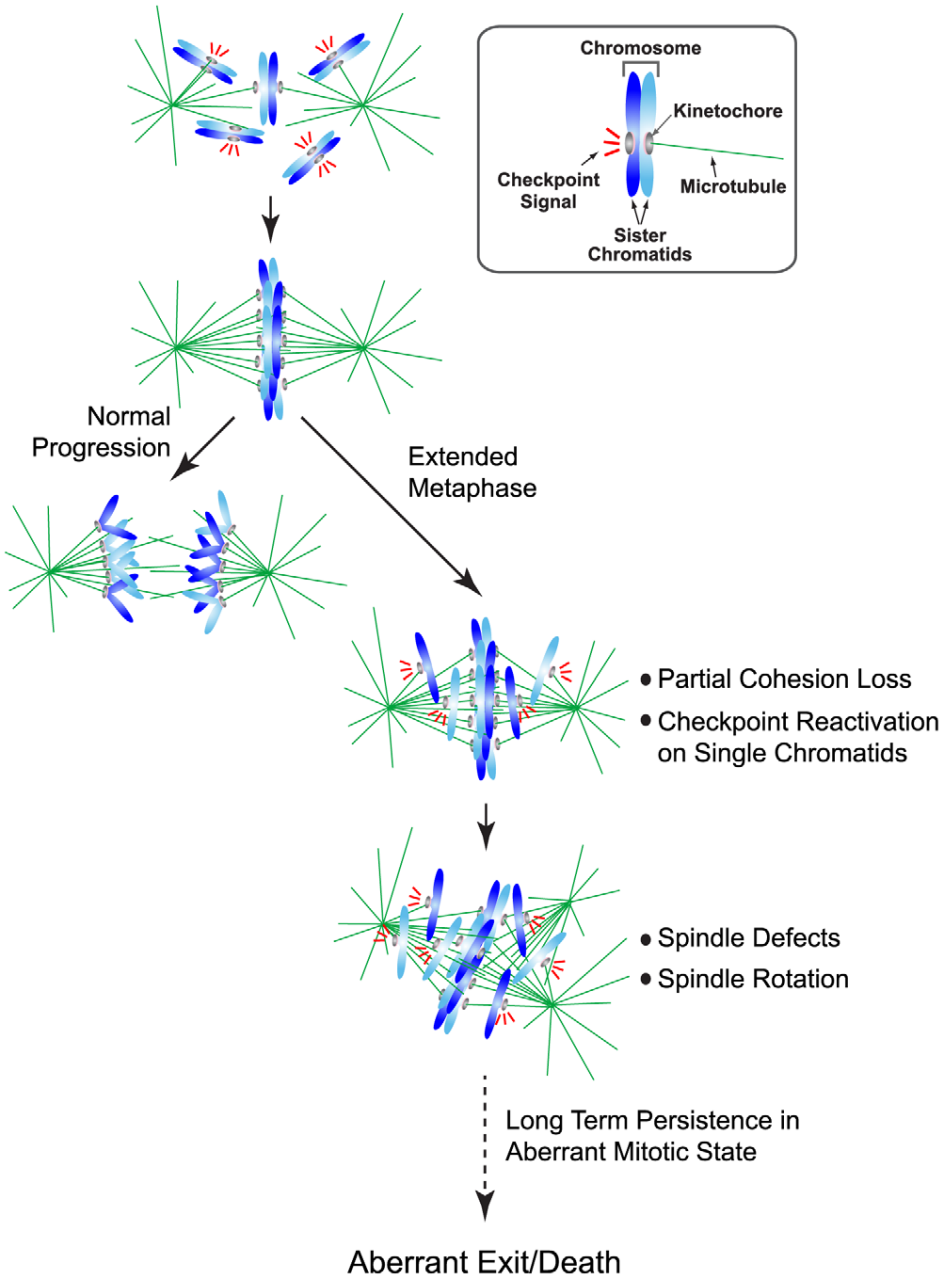
Model describing the chromosome scattering phenotype. In an unperturbed mitosis, sister chromatids align along the metaphase plate and lose their cohesion in a coordinated manner such that all sisters are separated simultaneously. In contrast, during a prolonged metaphase arrest, single unpaired sister chromatids escape from the plate while a majority of the chromosomes still remain paired. These single sister chromatids activate the spindle assembly checkpoint and hold the cell in an extended mitotic arrest during which further cohesion loss and spindle defects occur. Cells with scattered chromosomes remain in a long-term mitotic state and eventually escape via a non-anaphase like exit or cell death.

Both scattering and anaphase are associated with loss of chromatid cohesion. Anaphase onset is tightly coupled to biorientation of all chromosomes in the cell [27]. In contrast, scattering is observed with perturbations where all of the chromosomes are aligned as well as with perturbations where some chromosomes fail to align at the plate. Thus, it appears that too much time spent in a bioriented state initiates entry into the scattered state. Another key distinction between scattering and anaphase is that in the scattering phenotype, cohesion loss is partial and uncoordinated; a hallmark of anaphase is coordinated removal of cohesion that is coupled to Cdk1 inactivation. The lack of coordination in the presence of Cdk1 activity leads to reactivation of the spindle checkpoint on single chromatids following their dispersal from the plate. We propose that this traps the cell in an increasingly unstable mitotic state, leading to further cohesion loss (Fig. 7). The onset of scattering is also followed by spindle defects and spindle rotation within the cell (Fig. 7). At metaphase, biorientation of chromosomes and forces in the spindle are poised to coordinate separation of sister chromatids [28]. The partial loss of cohesion during scattering may lead to an imbalance in these forces and subsequent spindle structure defects.

The comparison between scattering and anaphase onset suggests that cohesion is a potential weak link during mitosis and rapid alignment and progression are important to avoid entering into the scattered state. It is currently unclear what features make a subset of chromosomes initiate scattering. Either a differential force depending on spindle location underlies susceptibility to scattering or there is chromosome-autonomous susceptibility, potentially dictated by the amount of cohesin present at individual centromeres.

### Mechanism of Scattering

There are two possible explanations for the dysregulation that leads to the partial uncoordinated cohesion loss that underlies scattering. The first explanation is that the separase-mediated cleavage reaction, which normally acts to trigger anaphase, is weakly activated in an extended mitotic arrest. The second possibility is that cohesion mediated by the cohesin ring is unable to mechanically resist microtubule-dependent pulling forces over an extended time. Prior work has suggested that weak separase activation may occur in nocodazole-arrested cells over time [29]. Using separase RNAi, we weakly delayed but did not inhibit the onset of scattering. However, this weak effect may reflect partial penetrance of the RNAi knockdown. The strongly synergistic suppression of scattering by Wapl RNAi and separase RNAi, as well as observation of centriole disengagement which involves separase activity [30], suggests that separase activation does indeed contribute to the scattering phenotype. However, this does not exclude the possibility that independently of cleavage, cohesin is unable to maintain cohesion over an extended period under mechanical load. A recent study similar to ours also reported partial loss of cohesion following extended metaphase arrest [31]. In that study, the authors proposed that cohesion fatigue induced by extended biorientation accounts for the loss of cohesion; however, neither they nor we can conclusively eliminate weak activation of normal cohesin removal pathways as the origin of the uncoordinated cohesion loss. Further work will be necessary to elucidate the mechanistic origins of the scattered phenotype.

### Implications of the Scattering Phenotype for Characterization of Mitotic Phenotypes

The prevalence of the scattering phenotype has significant implications for RNAi-based phenotypic analysis of mitotic mechanisms in human cells. Numerous knockdowns reported in the literature, when analyzed using fixed assays, show the hallmarks of this phenotype (e.g. [32]–[39]). As control cells treated in parallel do not show the phenotype, the observed defects are interpreted to reflect the function(s) of the targeted protein(s). Our observations suggest that future work must consider the possibility that defects observed in fixed assays may either reflect a function of the targeted protein or secondary consequences of entry into the scattered state. As scattering is observed with multiple perturbations that permit full or partial biorientation of chromosomes, many perturbations are likely to lead to eventual scattering. One approach that may help avoid confusion would be to perform live imaging from NEBD onward following knockdowns. In addition to revealing the primary phenotype, such an approach will also reveal if an extended metaphase arrest is occurring and whether this eventually results in scattering.

### Implications of the Scattering Phenotype for Evaluation and Design of Anti-Mitotic Agents

Anti-mitotic agents targeting tubulin are a mainstay of modern cancer chemotherapy. Increased mechanistic understanding of mitosis has led to significant interest and investment in the development of new highly specific anti-mitotic agents. The existence of the scattering phenotype in cells used to assess such agents has implications for these efforts. For example, recent work testing for the efficacy of taxol-mediated killing of cells suggested that cells held in metaphase by depletion of Cdc20 RNAi were more susceptible to taxol [21]. In that study, spindle defects were evident in the Cdc20 RNAi-mediated arrest but the chromosome behavior was not analyzed. We suggest that the Cdc20 RNAi may be causing these cells to enter the scattered state, and this in turn enhances their sensitivity to taxol. This example indicates how monitoring of entry into the scattered state may prove useful in evaluation of anti-mitotic agents.

A corollary to the above is the terminal nature of the scattered state and, in our comparison of HeLa FRT cells and hTERT-RPE1 cells, the significantly higher susceptibility of the former to scattering. This observation suggests that highly aneuploid cancer cells may be more susceptible to scattering than normal cells. Testing this idea will require high-resolution analysis of a large number of cell lines and quantitative analysis of their scattering properties using a uniform means for inducing metaphase arrest. If such an effort reveals that cancer cells show significantly higher susceptibility to scattering then development of therapeutic agents that induce scattering, potentially by holding cells in a metaphase-like state, may provide a new avenue for anti-mitotic chemotherapy.

## Materials and Methods

### Cell lines and drugs

Stable isogenic HeLa Flp-In T-Rex (HeLa FRT) cell lines expressing the nondegradable Cyclin B1 (lacking the N-terminal 86 amino acids) and the Spindly point mutant, both stably expressing H2b:mRFP, were previously generated [16], [40]. Stable expression of H2b:mRFP, and mRFP:H2b and YFP:α-tubulin, in hTERT-RPE1 and HeLa cells respectively, was done by retroviral delivery as previously described [41]. hTERT-RPE1 and HeLa cells were obtained from the ATCC (American Type Culture Collection, www.atcc.org). The CENP-E inhibitor used was a close analog of GSK923295 [19], [20] and was a gift from D. Cleveland and A. Shiau. For all experiments it was used at a final concentration of 1 µM. Reversine [23] was used at a final concentration of 0.5 µM. MG132 (Sigma) was used at a final concentration of 20 µM.

### Cell culture and RNAi

Cells were maintained at 37°C in a 5% CO_2_ atmosphere. HeLa cells were maintained in Dulbecco's modified Eagle's medium (Gibco), hTERT-RPE1 cells were maintained in DMEM/F12+Glutamax (Gibco) and both were supplemented with 10% tetracycline-free fetal bovine serum (Clontech), 100 U/mL penicillin and 100 U/mL streptomycin. For immunofluorescence, cells were seeded on 12-mm poly-L-lysine coated coverslips in 12-well plates 24 h prior to treatment. For live-cell imaging experiments, cells were seeded in a 35-mm glass-bottom dish coated with poly-D-lysine (MatTek). Cells were transfected using Oligofectamine and reduced-serum Opti-MEM (Invitrogen) according to manufacturer's instructions. A predesigned (Dharmacon/Thermo Scientific) siRNA for Spindly (GAAAGGGUCUCAAACUGAA), ESPL1/separase (GCUUGUGAUGCCAUCCUGAUU) or a nontargeting control (UGGUUUACAUGUCGACUAA) was used at a final concentration of 100 nM. ON-TARGETplus SMARTpool siRNA (Dharmacon/Thermo Scientific) was used for knockdown of Cdc20 (L-003225-00) and Wapl (L-026287-01) at a final concentration of 100 nM. After incubation for 5–6 hrs, 1 vol of medium and fetal bovine serum (10% final) was added. After 24 hrs, the transfection mixture was replaced with fresh medium. For live imaging of HeLa Flp-In T-Rex cells, transgene expression was induced with tetracycline (0.2 mg/mL) 24 hrs after transfection, and the filming session was initiated 8 hrs later.

### Live-cell imaging

For live-cell imaging, medium was replaced with CO_2_-independent medium (Gibco) supplemented with 10% tetracycline-free fetal bovine serum, 100 U/mL penicillin and 100 U/mL streptomycin. Tetracycline (0.2 mg/mL) was added to maintain transgene expression, and the medium was covered with mineral oil immediately before filming. Time-lapse images were recorded (every 4 minutes for the 10 hr filming, every 12 minutes for the 48 hr filming and every 30 sec for the 3 hr imaging) on a Deltavision microscope (Applied Precision) equipped with an environmental chamber heated to 37°C. Images were acquired with a CoolSnap charge-coupled device camera (Roper Scientific) and a 40× NA 1.35 U-planApo objective (Olympus) at 2×2 binning. Images were viewed and analyzed with MetaMorph software (Molecular Dynamics).

### Indirect Immunofluorescence

Cells were washed once with PBS and fixed with 4% paraformaldehyde in PBS for 10–15 min at room temperature, then permeabilized for 3–5 min with 0.1% Triton X-100 in PBS. Primary antibodies and dilutions used: ACA 1∶500 (Antibodies Incorporated), α-tubulin 1∶500 (DM1α – Sigma), Sas4 1∶500 (A. Dammermann, K. Oegema Lab), Mad1 1∶40 (a gift from A. Musacchio), Cyclin B 1∶100 (Santa Cruz) and securin 1∶100 (Abcam). Images were recorded on a Deltavision microscope at 1×1 binning with a 100× NA 1.3 U-planApo objective. Z-stacks (0.2-µm sections) were deconvolved using softWorRx (Applied Precision) and maximum intensity projections were imported into Adobe Photoshop CS4 (Adobe) for further processing. Determination of interkinetochore stretched was done as previously described [16].

## Supporting Information

Figure S1 **Cells with scattered chromosomes maintain the hallmarks of mitosis.** (**A, B, C**) Immunofluorescence images of control or scattered cells for (A and C) securin and (B) Cyclin B. For (A and B) the arrowhead indicates cells in metaphase, the arrow indicates cells in anaphase. In (C), the arrowhead indicates a scattered cell, the arrow indicates a cell that has entered an anaphase-like state. Scale bar, 5 µm.
(TIF)

Figure S2 **Immunoblot of protein inhibition via RNAi.** Whole cell lysates were probed for either separase or Wapl for the various conditions.
(TIF)

Movie S1 **Chromosome scattering results from perturbations that arrest cells in a metaphase-like state.** Movies start at NEBD. Time lapse is 4 min and playback speed is 1440×real time.
(MOV)

Movie S2 **Chromosome scattering results from perturbations that arrest cells in a metaphase-like state.** Movies start at NEBD. Time lapse is 4 min and playback speed is 1440×real time.
(MOV)

Movie S3 **Uncoordinated loss of cohesion and spindle defects are hallmarks of chromosome scattering.** Movie starts just prior to the onset of scattering with a fully aligned metaphase plate. Time lapse is 30 seconds and playback speed is 180×real time.
(MOV)
