# Supplementary figures and images for: Uncoordinated Loss of Chromatid Cohesion Is a Common Outcome of Extended Metaphase Arrest
Source: PLoS One. 2011 Aug 2;6(8):e22969. doi: 10.1371/journal.pone.0022969 (PMC3149067; doi:10.1371/journal.pone.0022969)

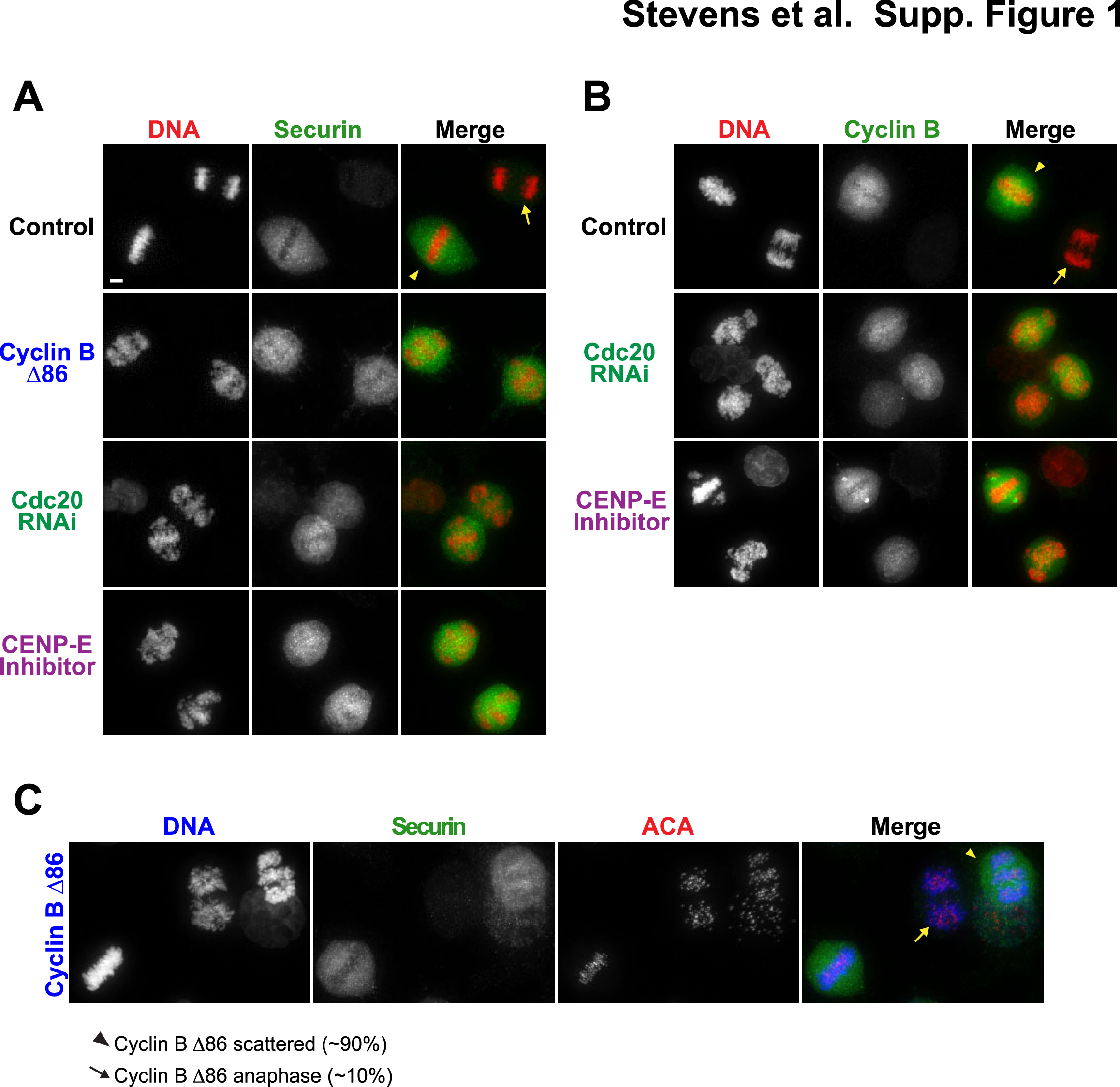

Supplement: Figure S1 — Cells with scattered chromosomes maintain the hallmarks of mitosis. (A, B, C) Immunofluorescence images of control or scattered cells for (A and C) securin and (B) Cyclin B. For (A and B) the arrowhead indicates cells in metaphase, the arrow indicates cells in anaphase. In (C), the arrowhead indicates a scattered cell, the arrow indicates a cell that has entered an anaphase-like state. Scale bar, 5 µm. (TIF) [file pone.0022969.s001.tif]

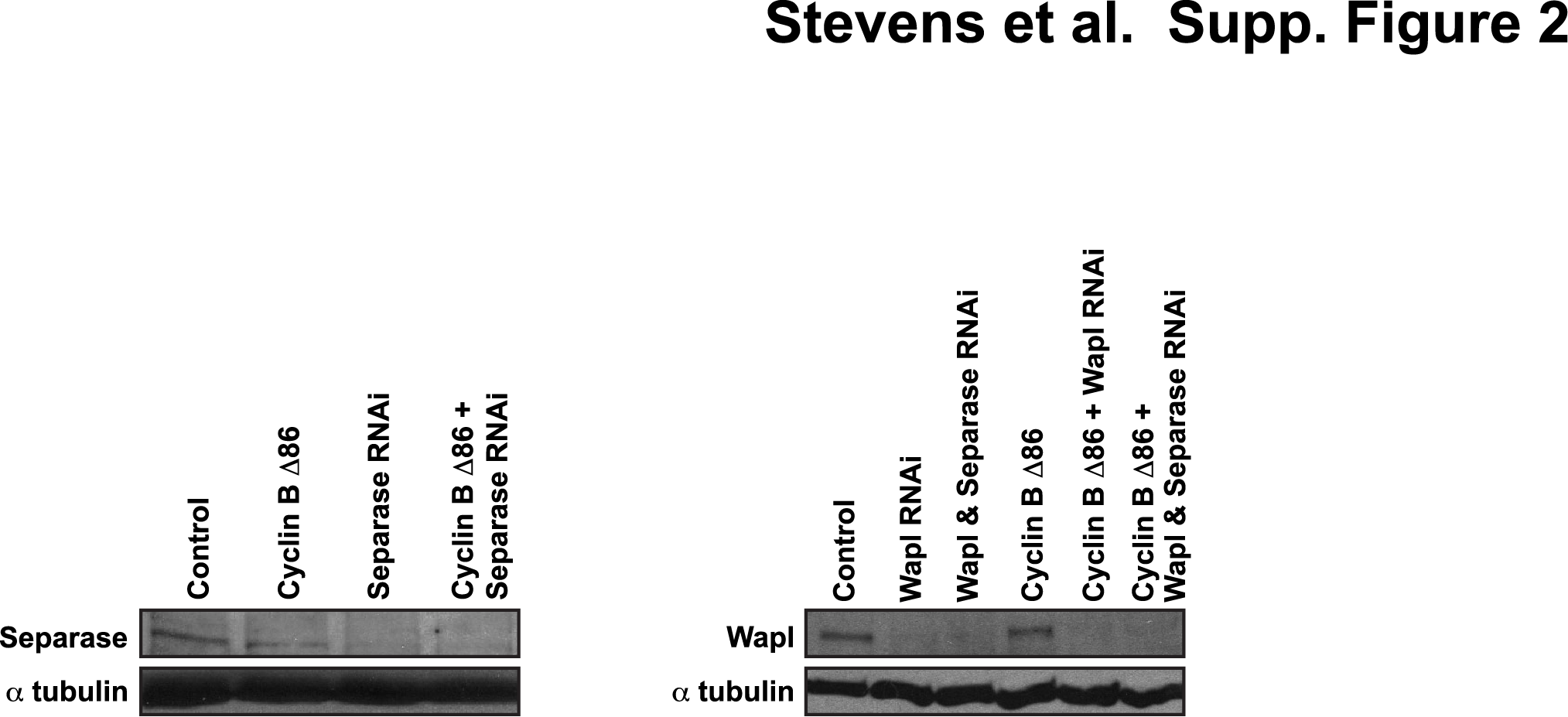

Supplement: Figure S2 — Immunoblot of protein inhibition via RNAi. Whole cell lysates were probed for either separase or Wapl for the various conditions. (TIF) [file pone.0022969.s002.tif]
